# Political polarization and public health in Germany

**DOI:** 10.3389/fpubh.2026.1925598

**Published:** 2026-10-19

**Authors:** Mesut Yavuz, Alexandra Roth, Nicolai Savaskan

**Affiliations:** 1 YES Automation, Nürnberg, Germany; 2 Department of Midwifery, University Lübeck, Institute of Health Sciences, Lübeck, Germany; 3 Public Health Authority Neukölln, Gesundheitsamt Neukölln, Berlin, Germany

**Keywords:** crisis response, health system reform, institutional design, Öffentlicher Gesundheitsdienst, political polarization, public health governance, public health resilience, self-governance

## Abstract

In contrast to its outpatient and inpatient healthcare, Germany’s public health service (Öffentlicher Gesundheitsdienst, ÖGD/PHS) faces financial and structural disadvantages. In this paper, we unravel the challenges associated with political polarization and their significant impact on public and individual health. Political polarization should be understood not merely as a communication challenge but as a governance determinant for public health. Using Germany’s public health service as an illustrative case, we examine how institutional dependence within political-administrative hierarchies can make the translation of scientific evidence into credible action more vulnerable under crisis conditions. Three mechanisms for scientific vulnerability are central: 1. the erosion of trust in institutions and expertise; 2. the rising political costs of acting in uncertain situations; and 3. reduced compliance when public health measures are interpreted through partisan lenses. Our investigations are based on a review of selected literature and institutional analysis focusing on the impact of political polarization on public health and subsequently discuss reform pathways that could strengthen scientific integrity, professional accountability, and crisis capacity, including statutory and professional safeguards for independence, tighter medical and academic integration of public health authorities, clearly defined leadership profiles, agency-based arrangements, and, in the longer term, self-governing models. At the same time, we emphasize trade-offs relation, legal oversight, financing, and federal coordination, as well as the limits of a non-causal, conceptually oriented argument.

## Highlights

- Political polarization should be considered a governance determinant of public health resilience, as it can undermine institutional trust, crisis decision-making, and compliance.
- In Germany, unlike self-governing healthcare sectors, the Public Health Service (ÖGD) remains embedded in governmental administration, making its scientific decisions more vulnerable to political pressures.
- Key mechanisms include erosion of trust, increased political costs of action under uncertainty, and reduced compliance when health measures are seen as politically influenced.
- Strengthening resilience requires not only resources but also institutional reform, e.g., legal/professional independence, stronger academic integration, defined leadership roles, agency models, or more autonomous governance.

## Introduction

According to the World Health Organization (WHO) (1–3), a resilient health system is characterized by health institutions and populations, that are “*able to effectively anticipate, prevent, prepare for, absorb and adapt in response to, and recover from a wide variety of shocks and stressors while delivering high-quality individual and population health services as needed, utilizing lessons from experiences within and outside their settings to continuously improve on their baseline capacities and performance in all contexts*” (4). The public health services must meet the same requirements at the population level. Public health resilience therefore has become a central concern of contemporary health governance, particularly in the aftermath of the COVID-19 pandemic and in light of increasingly complex societal crises. Pandemics, climate-related emergencies, demographic changes, and growing social fragmentation have demonstrated that the ability of public health systems responding effectively under pressure depends not only on operational capacity, but also on institutional trust, governance structures, and the credibility of evidence-based decision-making (5, 6). While resilience frameworks have evolved considerably over recent years, they continue to focus predominantly on technical preparedness, infrastructure, and resource allocation. Thus, shorting comings are insufficient attention to the political and institutional conditions that shape system performance during crises (7, 8).

In this study we suggest that political polarization should be understood as an important governance determinant of public health resilience. While the various dimensions of political polarization (such as affective, ideological, and elite polarization) interact, we refer here specifically to affective polarization, which we understand as sympathy for co-partisans coupled with rejection of opposite partisans (9). Its effects extend beyond political discourse alone and may influence interagency coordination and institutional trust (10) in the sense of a broad, deeply rooted confidence in institutions and public decision-makers. The loss of trust in turn, may lead to a deterioration of public compliance and the implementation of evidence-informed policy. In highly polarized environments, public health recommendations may increasingly be interpreted through partisan lenses, potentially undermining the perceived neutrality and legitimacy of public institutions. These dynamics became particularly visible during the COVID-19 pandemic, when scientific uncertainty, political contestation, and public communication challenges interacted in ways that affected the effectiveness and social acceptance of health measures across many countries (11).

Germany’s Public Health Service (Öffentlicher Gesundheitsdienst, abbreviated as ÖGD or PHS) provides an illustrative case through which to examine these questions. Unlike the self-governing health sectors for outpatients and inpatients services in the German healthcare system, the ÖGD remains institutionally embedded within governmental and administrative hierarchies at municipal and state level (12) (Figure 1). This structure reflects important principles of democratic accountability and administrative oversight, but it may also create tensions between medical and scientific independence (as defined by the European Commission) (13), professional responsibility, and political-administrative decision-making during periods of crisis. The pandemic exposed several long-standing structural challenges within the ÖGD, including workforce shortages, limited academic integration, fragmented digital infrastructures, and constraints on institutional visibility and authority (14).

**Figure 1:**
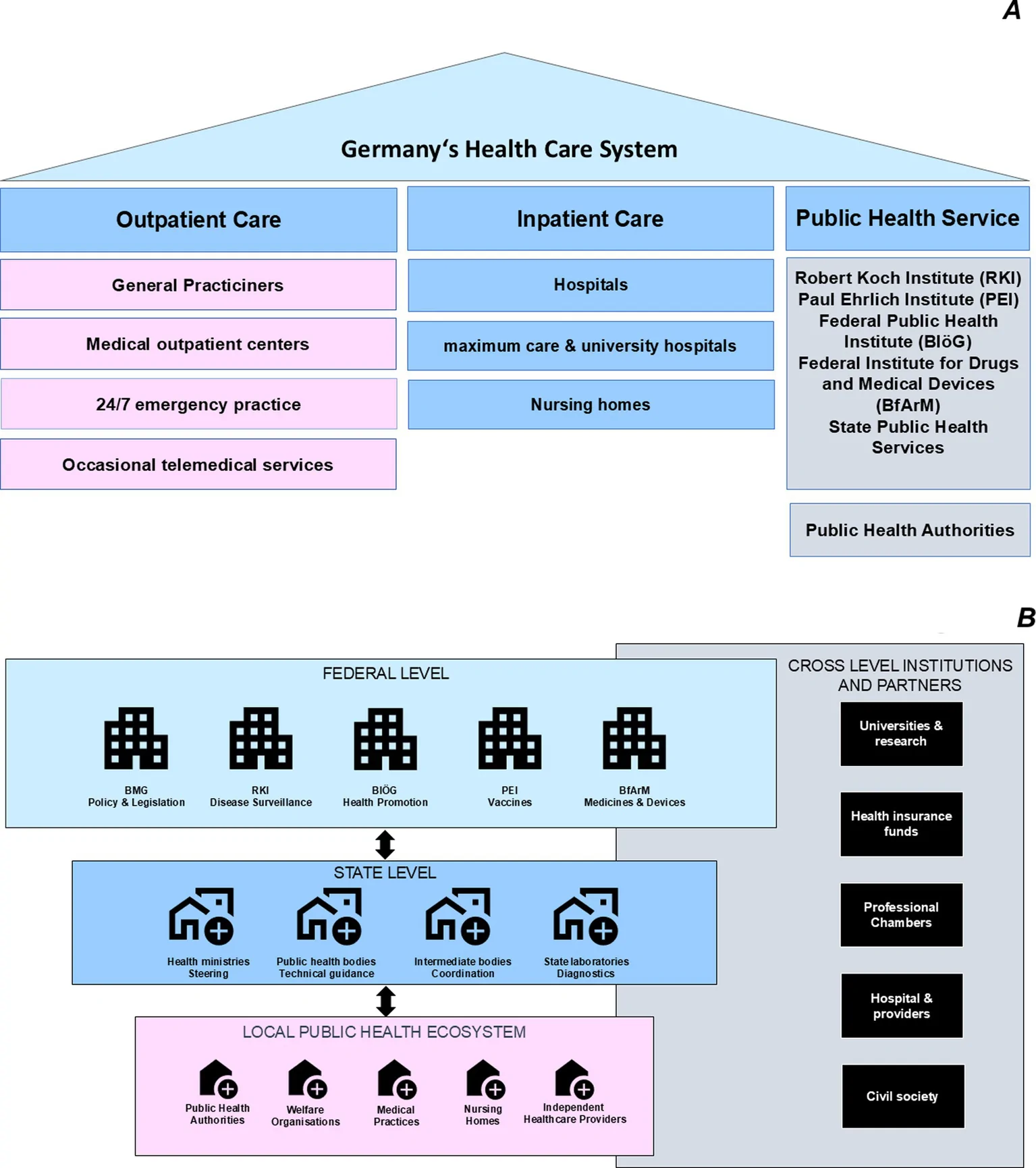
Germany’s health care system. Germany’s health care system is based on three columns. (**A)** a special feature of the public health service is its organization within the governmental institutions. Physicians act as medical service providers in three sectors, i.e., ambulatory, stationary and in the public administration service. Statutory health insurances cover mainly for the ambulatory and stationary medical care. **(B)** Institutional structure of the German Public Health Service and its wider public health ecosystem. Selected institutions and organizations are shown operating at the federal, state and local levels. Federal actors include the Federal Ministry of Health (BMG), the Robert Koch Institute (RKI), the Federal Institute of Public Health (BIÖG), the Paul Ehrlich Institute (PEI) and the Federal Institute for Drugs and Medical Devices (BfArM). At state level, responsibilities are shared between health ministries, public health institutions, regional or intermediate authorities and specialist laboratories. Their organization differs across the federal states. The local public health ecosystem includes local public health authorities, welfare organizations, medical practices, nursing homes and independent healthcare providers. The arrows indicate coordination and information exchange between the administrative levels rather than a uniform chain of command. The organizations shown on the right contribute across more than one level. This graph display a simplified structure, as institutional arrangements vary across Germany’s federal states.

The purpose of this article is not to present causal proof of primary empirical findings, but to offer a conceptual perspective on how governance structures and political polarization may interact to shape public health in crisis and resilience. Drawing on literature, we examine how political polarization affects the implementation and acceptance of measures by public health services. In a subsequent step, we link these findings to the institutional experience of the German governmental public health service (ÖGD). Our aim is to explore how political polarization can amplify existing structural vulnerabilities. Furthermore, we present options of possible reform pathways aimed at strengthening scientific integrity, professional and medical autonomy, and institutional trust. These include, among others, stronger statutory safeguards for professional independence, closer integration between public health practice and academia, clearer leadership and qualification structures, agency-based governance models, and more autonomous forms of institutional organization.

By framing resilience not only as an operational challenge but also as a question of institutional mindset design and governance capacity, this Perspective seeks to contribute to broader debates on the future organization of public health service in politically and socially contested environments.

## Methods and data resources

In the present study we employed a mixed-methods approach, integrating elements of a scoping review, data-based citation, and thematic analysis mapping the current literature. This approach ensures a broad yet in-depth exploration of scientific literature as well as gray literature. This approach minimizes the risk of missing key points and providing a robust synthesis of the existing knowledge base. A preliminary scoping review was based on the World Health Organization’s strategic and policy documents. This provided an overview of the subject and ensures that an extensive search is conducted in the initial stage. The inclusion criteria encompassed references to public health service, governmental and policy papers, and published interviews of leading persons of the german administration.

## Structural vulnerability in context: the German public health service

Germany’s Public Health Service (Öffentlicher Gesundheitsdienst, ÖGD or PHS) provides a particularly instructive case study through which these relationships can be examined—one that combines institutional specificity with broader structural lessons of considerable comparative relevance. Within the architecture of the German healthcare system (15, 16), the public health service (PHS) occupies a position that is structurally anomalous and historically contingent.

The outpatient and inpatient sectors together constitute the dominant axes of healthcare delivery. They predominantly treat patients insured by the statutory health insurance (SHI), while the private health insurances (PHI) play a minor role in Germany. Patient care within the SHI system is based on the principles of solidarity and benefits in kind, and are organized through elaborate mechanisms of self-governance (Selbstverwaltung) (17). The federal government sets the framework for health care. Within this framework, professional associations, insurers, and providers exercise substantial regulatory authority with meaningful independence from direct state control (Figure 1B).

Public health authorities, by contrast, remain embedded within governmental administration at the federal, state and municipal levels—accountable to elected officials and subject to the hierarchical imperatives of the administrative state (18). On the national level there are several institutions with overarching areas of responsibility such as infection control, health communication, and the safety of food, medicines and vaccines (Figure 1). Since the health care system in Germany is organized on a federal basis such as insurances, investments in infrastructure and regulations, there are also state health ministries and agencies with impact at the state level. Finally, at the local level, there are the subordinate public health authorities, which enforce all laws and regulations enacted at the federal and state levels, and carry out a wide range of tasks related to public health protection and prevention for the local population (17).

This arrangement is not accidental. It reflects a deeply rooted institutional trajectory shaped by, at that time West-Germanys post-war commitments to democratic accountability and the constitutional principles that state functions bearing direct societal consequences must remain subject to political and parliamentary oversight. These are not trivial considerations. Yet the same structural logic that furnishes democratic legitimacy simultaneously generates a dependency on political officeholder that carries significant costs. By embedding public health authorities within governmental hierarchies rather than granting them the professional autonomy afforded to other sectors of the health system, the institutional design creates conditions in which scientific assessments may be filtered through administrative and political channels. As a result, professional autonomy may be constrained by organizational loyalty, and the boundaries between evidence-based counsel and politically palatable output may become dangerously blurred. This becomes most acute precisely in those moments of crisis when the integrity of that boundary is crucial.

The COVID-19 pandemic brought these latent tensions into sharp and unsparing focus. German public health authorities were required, often simultaneously, to navigate conditions of unprecedented scientific uncertainty, interpret and communicate rapidly evolving epidemiological evidence to heterogeneous audiences, and maintain operational coherence under the glare of intense and frequently adversarial political scrutiny. The demands were extraordinary; the structural foundations on which authorities were expected to meet them were, in critical respects, inadequate. Pre-existing limitations—chronic workforce shortages, a comparatively low proportion of medically trained personnel within the ÖGD, fragmented integration between public health practice and both the broader healthcare system and academic research institutions—significantly constrained the capacity of authorities to respond with the speed, consistency, and analytical depth that the crisis required (19). These were not deficiencies that emergency measures could easily overcome; they were the accumulated expression of decades of institutional underinvestment and structural neglect (20). More troubling still, empirical observations from the pandemic period suggest that, in certain documented instances, scientific assessments were subordinated to political considerations—that the conclusions reached by public health professionals were shaped, filtered, or delayed by the administrative and political hierarchies within which those professionals operated (21). Such episodes provoked legitimate and serious concerns about the functional independence of public health decision-making and the long-term credibility of the institutions responsible for it. It would be analytically insufficient, however, to treat these developments as isolated failures of individual judgment or organizational mismanagement. They are more accurately understood as the predictable manifestations of underlying institutional configurations—structural arrangements that, under ordinary conditions, may remain latent and manageable, but that under conditions of acute crisis become not merely visible but consequential in ways that can determine the course of events.

## Political polarization as a force multiplier: from amplifier to structural determinant

Political polarization does not merely coexist with the institutional vulnerabilities. In addition, political polarization actively amplifies them, introducing additional and compounding layers of complexity into governance processes that are already operating under conditions of structural strain. Its most immediate effect is a category transformation: through polarization, questions that are fundamentally technical or scientific in nature are converted into objects of ideological contestation, stripped of their epistemic authority and recast as positions in a broader cultural or partisan conflict. The consequences for public health governance are severe. Decision-making processes become encumbered by the need to anticipate and manage political reactions, often at the expense of scientific clarity. Public health messaging, which derives its effectiveness from trust and consistency, precision, and perceived impartiality, is fatally compromised when the institutions delivering it are perceived as partisan actors and moral loss rather than neutral arbiters of evidence (Figure 2A). In polarized environments, the content of a public health recommendation matters less than its source: measures endorsed by institutions that one political constituency trusts are, by that same logic, regarded with suspicion or outright rejection by another (22). Compliance diminishes not as a function of the measure’s rationality but as a function of its perceived ideological valence. This dynamic is especially pronounced in local rural settings, where the conditions for its propagation are structurally embedded. The lack of social diversity in remote rural areas with low economic strength is associated with increased support for right-wing parties, while greater social diversity among disadvantaged and privileged groups in urban neighborhoods appears to counteract polarization. At the same time, there was consistent evidence of differences in vaccination willingness between rural and urban settings during the COVID-19 pandemic. In the U.S., those who were more skeptical of vaccination placed greater trust in the opinions of family and friends than those willing to be vaccinated, who primarily trusted doctors and health professionals (23, 24) (Figure 2A). Furthermore, the extent of vaccine skepticism in the U.S. was positively correlated with lower educational attainment and a higher proportion of Trump supporters (23, 24). In Germany, spatial data are not available nationwide due to the lack of a vaccination registry. However, a 2021 survey found a strong correlation between an individual’s willingness to get vaccinated and that of their social network, as well as significantly lower trust in the leading national public health institute, the Robert Koch Institute (RKI), among vaccine skeptics (25).

**Figure 2:**
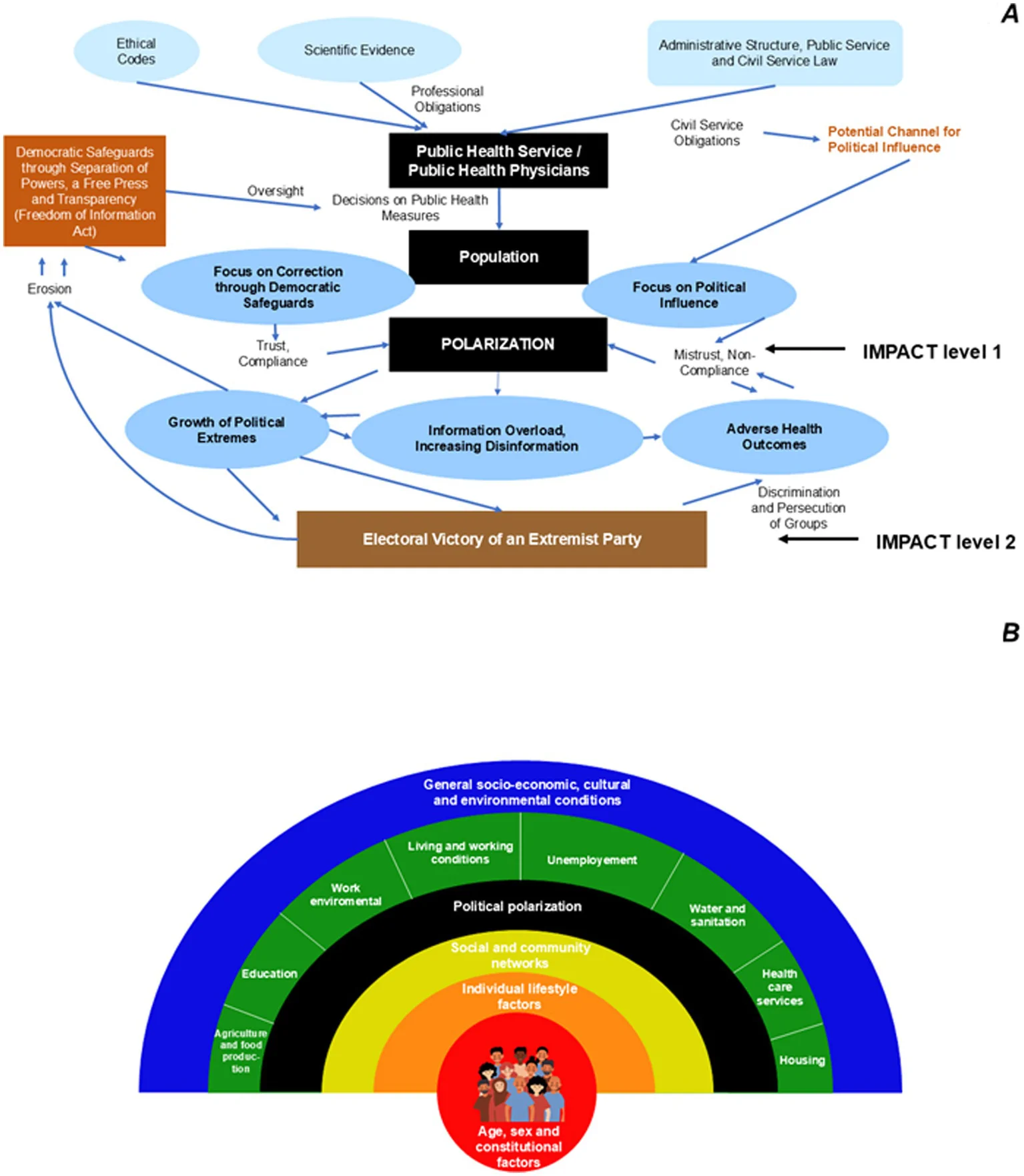
Current determinants of health. **(A)** The interaction network of political polarization and the level of impact on public health service (ÖGD). **(B)** This graph displays an extended rainbow model of determinants of health inequities based on the original Dahlgren and Whitehead model (30). In addition to general socio-economic, cultural and environmental conditions there are evidence to theorize and operationalize political polarization as an independent determinant of health inequities.

The city as well as small rural communities, in this sense, are not merely a site where polarization has public health consequences, it is a terrain that actively accelerates and intensifies health inequities (26–28). Against this backdrop, a conceptual reorientation seems not merely sensible but analytically necessary: in our view, political polarization must, by itself, be reconceptualized as a structural determinant of public health resilience. Accordingly, it should be given the same systematic attention that that has traditionally been accorded to the established determinants of health such as socioeconomic conditions, environmental exposures, and individual behavioral risk factors. (26, 29, 30). Traditional models of health determinants have generated indispensable insights, and their explanatory power with respect to population health outcomes remains substantial and well-evidenced. Yet, these frameworks were developed to account for a particular register of causation, one in which health outcomes are understood principally as functions of material circumstance, biological exposure, and personal agency (Figure 2B). They are structurally ill-suited to capture the institutional and political dimensions that shape system performance at the level of governance. Their silence on these dimensions no longer seems defensible in an era defined by the systemic consequences of a loss of trust in public institutions. By incorporating political polarization as a further determinant of health into the framework of Dahlgren and Whiteheads, it becomes possible to account for mechanisms that conventional models render invisible. An expanded model makes it possible to better identify three dimensions of the effects of political polarization:

1. the ways in which governance fragmentation distorts the translation of evidence into policy,
2. the manner in which institutional delegitimization reduces the population-level reach of public health interventions, and.
3. the processes through which polarized political environments and artificial intelligence-driven social media transform the credibility of public health authorities from a durable asset into a contingent and precarious resource (31, 32). In our view, such an inclusion significantly expands the analytical scope that research must cover to ensure social and medical resilience (29).

## Historical legacies, ethics, and the limits of institutional capacity

Germany’s experience gives this analytical perspective a particular historical weight and ethical urgency that transcends the merely theoretical. The institutional development of the PHS cannot be understood in abstraction from the darkest chapter of its history: the systematic instrumentalization of public health structures during the National Socialist period, when medical and public health institutions were co-opted in service of a state apparatus committed to racial and political classification, forced sterilization, and ultimately genocidal violence (33). This history is not an embarrassing prolog to be acknowledged and set aside. It constitutes, rather, a foundational reference point for every subsequent debate about the appropriate relationship between public health institutions and political authority, as a permanent reminder of what becomes possible when professional autonomy in conjunction with Prussian tradition of deference to authority in public administration is subordinated to the imperatives of the state and when the ethical obligations of public health practice are dissolved into administrative obedience (34). The Lancet Commission on medicine, Nazism, and the Holocaust has documented in exacting detail how professional structures that lacked meaningful independence from political direction proved incapable of resisting institutional capture (35). The lesson was not lost on the architects of the post-war German healthcare system. Following the Second World War, deliberate and consequential efforts were made to establish robust self-governance mechanisms across large parts of the healthcare landscape with arrangements designed precisely to insulate professional practice from direct state control and to distribute regulatory authority across a plurality of actors. Yet, public health authorities were not afforded equivalent structural protection (Figure 1). They remained embedded within governmental and administrative hierarchies of the civil service law, subject to the authority of elected officials and the disciplinary logic of the Prussian tradition of public administration (36, 37). The result is a structural asymmetry between the self-governing sectors of the healthcare system for inpatients and outpatients, on the one hand, and the administratively subordinate public health service, on the other. This imbalance has never been fully eliminated and persists to this day, along with all its attendant vulnerabilities (38, 39).

This structural asymmetry between PHS and ambulatory and stationary health care carries profound implications for professional autonomy and the lived practice of public health ethics (39). Such political dependence is manifested in the daily conditions under which public health professionals have to take decisions. Practitioners within the PHS operate within an institutional framework that demands a constantly, and often challenging, balancing act between two distinct sources of authority: the evidence-based obligations of scientific practice and the administrative directives of governmental hierarchies to which they remain formally accountable. Under ordinary conditions, this tension may be managed through institutional convention, professional discretion, and the tacit accommodations that characterize all complex bureaucratic environments. Under conditions of crisis or political pressure, however, it becomes acute, and the accommodations that ordinarily contain it begin to fail. The ethical stakes are considerable. Professional standards of the highest standing, most notably those articulated in the Internation code of medical ethics (Declaration of Geneva) (40), impose on medical and public health practitioners an unambiguous obligation to act in the best interests of patients and populations, independent of institutional convenience or political expediency. These are not aspirational commitments; they constitute the ethical bedrock upon which the social legitimacy of the medical and health profession rests. Yet, within hierarchical systems structured around administrative accountability and organizational loyalty, the practical capacity to honor these principles may be systematically constrained—not through explicit coercion, but through the subtler disciplinary mechanisms of institutional dependency: career vulnerability, reputational risk, and the internalized anticipation of organizational consequences. In polarized political environments, these pressures intensify even further. When public health decisions become objects of partisan contestation, when professional recommendations are subjected to public interrogation framed in ideological rather than scientific terms, and when the institutional affiliation of a practitioner becomes itself a target of political critique, the space available for the exercise of independent professional judgment shrinks quietly and incrementally. The consequences may not become fully visible until the crisis situations arise, when that judgment is needed most urgently.

The institutional vulnerabilities outlined above are further compounded by structural constraints in workforce composition and systemic capacity in Germany’s PHS that have accumulated over decades of relative neglect. No emergency mobilization can rapidly reverse these constraints. The PHS workforce was expanded during the acute phases of the COVID-19 pandemic: a response that reflected both the urgency of the situation and the political attention it received. Yet, this expansion concealed as much as it revealed. The proportion of medically trained personnel within the service remains comparatively low relative to equivalent public health structures in other high-income countries, reflecting longstanding deficits in the attractiveness of public health as a professional destination (43). Recruitment continues to be structurally inhibited by limited and rigidly defined career pathways, which put public health experts with scientific backgrounds at a disadvantage compared to candidates with general administrative training. Furthermore, physicians’ remuneration fails to compete with the financial incentives offered in the outpatient and inpatient sectors. Finally, due to a persistent status gap, work in the PHS is perceived as more administratively oriented than medically or intellectually demanding. Such perception discourages precisely the highly qualified professionals whose analytical capabilities the PHS most urgently requires. Compounding these workforce deficiencies is the weak and largely underdeveloped integration between public health practice and academic research institutions (41). In systems where this integration is strong, the boundary between practice and research becomes productively permeable: practitioners draw on current evidence, researchers orient their questions toward operationally relevant problems, and the knowledge base of the system is continuously renewed through mechanisms of structured feedback. In the German public health service, this relationship remains attenuated, limiting the development and systematic application of evidence-based approaches at precisely the moments when adaptive, analytically science-based responses are most needed (42). Taken together, these limitations substantially reduce the system’s capacity to recalibrate, innovate, and maintain analytical rigor in the face of complex, rapidly evolving, and politically charged crises, rather than merely execute established protocols tailored to familiar conditions.

## Toward structural reform: institutional architecture as the foundation of resilience

Addressing these deeply rooted challenges demands a fundamental reorientation of reform ambition. We propose a shift in analytical focus from the operational improvements that have dominated policy discourse so far to the structural transformations without which those improvements will remain insufficient. Investments in digital infrastructure, pandemic preparedness logistics, and workforce expansion are necessary and must be pursued with urgency. The pandemic exposed real and consequential gaps at each of these levels that cannot responsibly be left unaddressed (43). However, in our view treating such investments as the primary or ultimate objective of the reform means misjudging the nature of the problem. Operational capacity deployed within institutionally compromised structures does not produce resilient systems but produces systems that are better equipped to fail in more sophisticated ways. In our view and in the view of Lothar Wieler (former president of the Robert-Kich Institute, RKI), the fundamental problem lies not in resources, but in the institutional architecture (44). We therefore see a need for action to structurally guarantee scientific independence within the PHS, to protect professional autonomy from administrative and political interference, and ensure that the authority of evidence is not routinely subordinated to the imperatives of governance hierarchies. In this regard, the transformation of public health authorities into self-governed public corporations, a model with established precedent in other domains of the German healthcare system, may represent a structurally coherent and analytically compelling pathway for reform (34, 45). This approach would not dissolve the legitimate demands of democratic accountability. Rather, it would rebalance them against the equally legitimate demands of professional self-regulation. Furthermore it would establish institutional arrangements in which scientific integrity is protected by design rather than the goodwill of administrative superiors. The result would be a governance architecture better positioned to withstand the pressures of political polarization, to maintain the credibility of public health institutions in contested environments, and to sustain the conditions under which genuine resilience becomes structurally achievable.

From a systemic perspective, the preceding analysis converges on a conclusion that is both analytically robust and practically consequential: resilience is not, at its foundation, a function of resources or preparedness, although those dimensions display also important factors. It is, more precisely, a function of the conditions under which decisions are made, communicated, and implemented—conditions that are themselves the product of institutional design rather than circumstantial fortune. This distinction matters enormously for how reforms are conceived and prioritized. A system may possess abundant material resources, sophisticated surveillance infrastructure, and meticulously rehearsed emergency protocols, and yet remain profoundly fragile if the institutional conditions governing its decision-making are compromised. Conversely, a system operating under resource constraints may nonetheless demonstrate remarkable resilience if its governance architecture preserves the integrity of scientific deliberation, shields professional judgment from political interference, and sustains the social trust on which population-level compliance ultimately depends. Institutions that are structurally dependent on political authority face a distinctive and recurring vulnerability: under conditions of normalcy, this dependency may impose only modest costs, absorbed through informal accommodations and the relative stability of the political environment. Under conditions of crisis—precisely when the coherence, consistency, and perceived impartiality of public health institutions are most consequential—the same dependency becomes a source of systemic fragility, as institutional credibility is drawn into the contested terrain of political conflict and eroded by the very pressures that demand its preservation. The converse proposition is equally important: systems that incorporate robust, structurally embedded mechanisms to protect scientific integrity and professional autonomy are not merely better governed in an abstract sense—they are demonstrably better positioned to respond to crises with the consistency, authority, and adaptability that effective public health action requires. Resilience, understood in these terms, is an institutional achievement before it is an operational one.

The implications for public health resilience extend well beyond the specifics of the German case to raise fundamental questions about the design of public health systems in diverse national and institutional contexts. Cities are not merely the settings in which public health crises unfold—they are the primary terrain on which the adequacy or inadequacy of governance arrangements is tested, exposed, and judged by the populations who depend on them. As urban environments confront an increasingly dense and overlapping constellation of challenges—pandemic threats, climate-driven health emergencies, deepening socio-economic inequalities, and the accelerating fragmentation of shared epistemic frameworks—resilience strategies that focus exclusively on technical preparedness will prove systematically inadequate. What is in addition required is a new generation of governance frameworks that treat institutional independence, civil participation scientific credibility, and public trust not as desirable qualities to be aspired to, but as structural assets to be actively designed, protected, and renewed (46). This demands, in practical terms, that health planners, policymakers, and international health organizations incorporate governance risk assessments into their resilience planning, develop metrics for institutional credibility alongside those for operational capacity, and invest in the legislative and regulatory architectures that protect public health authorities from political capture. It further requires comparative learning across jurisdictions—an honest reckoning with the governance failures that the pandemic exposed and a commitment to institutional innovation that matches the scale and urgency of the challenges that lie ahead.

## Conclusion

The argument advanced in this paper admits of a clear and consequential conclusion: political polarization constitutes a critical, yet systemically under-recognized, determinant of public and individual health. In particular the PHS could be influenced through political polarization since PHS does not operate at the margins of system performance but at its institutional core. The German PHS examined here as both an empirical case and an analytical lens, illustrates with clarity how institutional design functions as a double-edged inheritance: capable, under favorable conditions, of providing the administrative coherence and democratic legitimacy that effective public health governance requires, yet equally capable, under conditions of political pressure and structural strain, of constraining professional autonomy, filtering scientific judgment, and eroding the credibility on which public health authority ultimately depends. To strengthen resilience, therefore, is not simply to invest in preparedness—it is to subject the political and institutional architectures of public health systems to the same critical scrutiny that the field has long applied to epidemiological risk and logistical capacity. This demands a broader analytical approach, one capacious enough to encompass not only the operational and technical dimensions of system performance but the governance environments in which that performance is embedded and from which it cannot, in practice, be separated.

Future strategies for public health resilience should move decisively beyond the narrow and increasingly inadequate conceptions of preparedness that have dominated policy discourse, and commit to a substantive agenda of institutional reform. This requires concrete measures to enhance the structural independence of public health authorities. With this the integrity of scientific deliberation could be protected from political encroachment, and ensure that the credibility of public health decision-making is treated as a strategic asset requiring active and sustained investment rather than a passive quality assumed to persist in the absence of deliberate maintenance. The challenges ahead—epidemiological, climatic, social, and political—will not diminish in complexity or intensity. By addressing these foundational institutional elements, and by recognizing political polarization as a structural threat to public health resilience rather than a background condition to be endured, public health systems could sustain the effectiveness, legitimacy, and public trust that their essential social function demands.

## Data Availability

The original contributions presented in the study are included in the article/supplementary material, further inquiries can be directed to the corresponding author/s.
